# Development and Application of the Anti-High-Temperature Delayed Crosslinking Polymer as a Gel Plugging Additive for Drilling Fluid

**DOI:** 10.3390/gels10010073

**Published:** 2024-01-18

**Authors:** Quanyang Wang, Jing Cai, Jiannan Wang, Chenghua Zhou, Xinxin Wen, Jiang Zhang, Hui Mao

**Affiliations:** 1 Drilling Engineering Research Institute, Sinopec Xinan Oilfield Service Corporation, Deyang 618000, China; wangqy.osxn@sinopec.com (Q.W.);; 2 College of Energy Resources, Chengdu University of Technology, Chengdu 610059, China; 201704010427@stu.cdut.edu.cn (X.W.);

**Keywords:** well leakage, plugging material, polymer gel, temperature resistance, delayed crosslinking

## Abstract

With the gradual deepening of the exploration and development of deep and ultra-deep oil and gas resources, the problem of lost circulation in drilling operations is becoming more and more complex. From field experience, conventional plugging materials cannot fully meet the technical requirements of plugging operations in drilling engineering. In this study, a high-temperature- and salt-resistant polymer HDZ−A was synthesized. A high-temperature and delayed crosslinking polymer gel plugging agent can be prepared by adding a certain concentration of a crosslinking agent and a retarder. In this paper, the optimum synthesis conditions of the HDZ−A were determined with orthogonal experiments using viscoelasticity and viscosity as evaluation criteria for newly developed polymers. The molecular structure, temperature resistance, and relative molecular mass of HDZ−A were determined using infrared spectroscopy, nuclear magnetic resonance spectroscopy, and gel permeation chromatography. In addition, the optimal formula of the gel plugging agent was determined using gel strength as the evaluation standard. The results show that the newly developed gel plugging agent has stable performance after high-temperature crosslinking, and can resist high temperatures of 160 °C during formation. Under conditions of 160 °C, the gelation time can reach 4.5 h, and the plugging efficiency can reach more than 97%. Finally, the field test of the newly developed high-temperature-resistant delayed crosslinking polymer gel plugging agent was carried out in the direct exploration well KT-14X in the Ordos Basin. The field test showed that the plugging effect of the HDZ−A gel plugging agent was remarkable.

## 1. Introduction

As oil and gas exploration and development deepen, the cost of well leakage treatment has significantly increased [1,2,3,4]. Formation leakage not only affects drilling efficiency but also leads to issues, such as unstable wellbores and even the blockage of permeable channels, resulting in reduced oil well production [4,5,6,7,8,9]. Currently, the plugging materials primarily used in drilling sites can reduce losses caused by well leakage, but their effectiveness is unsatisfactory when dealing with crack-type and cavern-type leakages [10,11,12,13]. Cement slurry is commonly used for such leakages, but it is easily diluted and washed away by formation water, making it difficult for it to stay in the near-well leakage zone [9,11,14,15,16,17,18].

Polymer gel plugging materials are also commonly used for plugging [19,20,21], as long as the gelation time can be precisely controlled and solidification occurs rapidly after reaching the target formation, overcoming the issue of staying in the formation [22,23,24]. However, polymer materials currently used for plugging still have limitations [9,18,25,26,27]. The control of polymer crosslinking time and the strength of the resulting gel after crosslinking is not easy [28,29], and there are also some deficiencies in terms of temperature resistance and salt tolerance [30].

Therefore, in this study, to address the shortcomings in delayed crosslinking and temperature resistance, a salt-tolerant polymer, HDZ−A, was synthesized using monomers such as polyacrylamide as raw materials. With HDZ−A as the key treatment agent, a crosslinking agent that can delay and control the crosslinking time was carefully selected, resulting in the development of a polymer gel plugging system that can withstand 160 °C and has a delayed crosslinking time of up to 4.5 h.

## 2. Results and Discussion

### 2.1. Orthogonal Test

An orthogonal experiment with four factors and three levels was designed. Table 1 is the factor level table of the orthogonal experiment.

Table 2 presents the orthogonal experimental design for synthesizing HDZ-A. The synthesized polymer was subjected to rheological and viscoelastic testing. Based on the results, the factors influencing the comprehensive performance of the polymer synthesis reaction were ranked in the following order: monomer molar ratio > reactant concentration > temperature > initiator mass fraction. According to the analysis of the mean values, the optimal synthesis conditions were determined as A1, B1, C3 and D3. This corresponds to a monomer concentration of 8 wt. %, an initiator mass fraction of 0.3 wt. %, a temperature of 70 °C, and a monomer molar ratio of AM:AMPS:ACMO = 7:1.75:1.25.

### 2.2. Characterization of Structure and Micromorphology

#### 2.2.1. FT−IR

**Figure 1:**
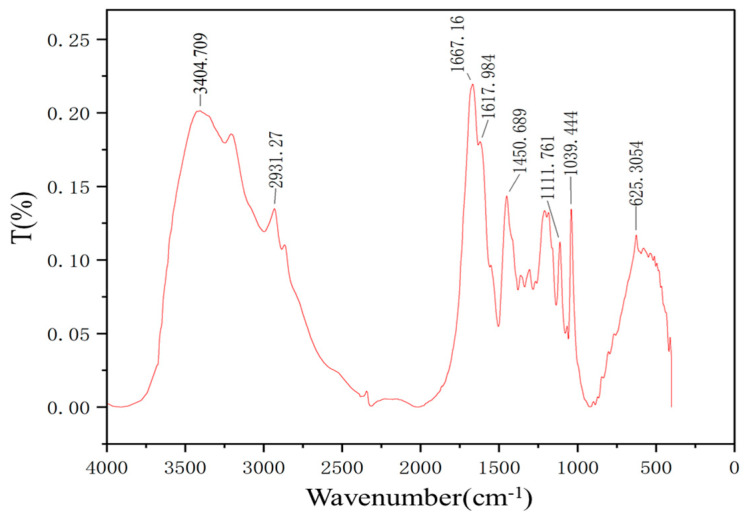
FT−IR image of HDZ−A.

From the infrared spectroscopy results shown in Figure 1, it can be observed that the characteristic absorption peaks are as follows: The peak at 3404 cm^−1^ corresponds to the characteristic absorption of free −NH_2_ groups. The peak at 2931 cm^−1^ corresponds to the asymmetric stretching vibration of methylene groups. The peak at 1617 cm^−1^ corresponds to the bending vibration of the amide group (N−H). The peak at 1677 cm^−1^ represents the characteristic absorption of the amide group, corresponding to the stretching vibration of the amide bond (C=O), originating from the main chain of acrylamide and AMPS. The peak at 1450 cm^−1^ corresponds to the characteristic absorption of methyl groups. The peak at 1111 cm^−1^ corresponds to the stretching vibration of the C−N bond, representing the morpholine group from the N−acryloylmorpholine monomer. The peak at 1039 cm^−1^ is related to the −SO_3_H group, originating from the AMPS monomer. The presence of sulfonic acid groups, cyanide groups, and other temperature-resistant functional groups indicates that the synthesized product exhibits high-temperature resistance. The FTIR characterization results confirm the successful synthesis of the target product.

#### 2.2.2. TGA Analysis

**Figure 2:**
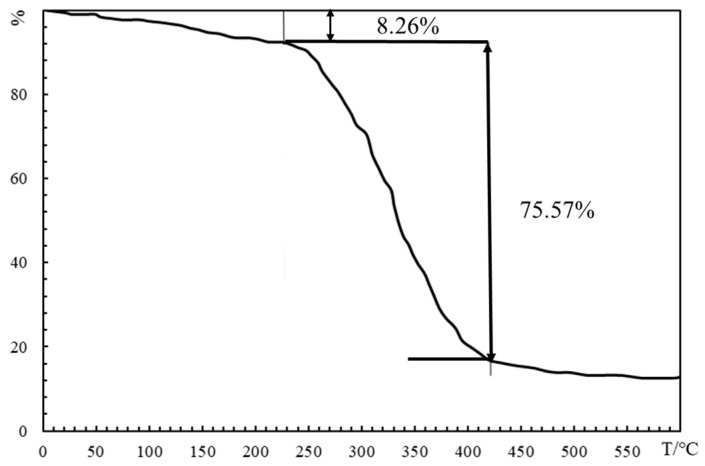
Thermogravimetric analysis curve.

Figure 2 presents the thermogravimetric (TG) curve of HDZ−A, providing the following information:

Within the temperature range of 0 to 240 °C, the polymer experiences a mass loss of 8.26%. This loss is primarily attributed to the evaporation of water and the volatilization of unreacted small molecules. In the temperature range of 240 to 430 °C, there is a sharp decrease in the TG curve, indicating a significant mass loss of 83.83% for HDZ−A. This suggests that the polymer’s network structure is disrupted, and the macromolecular chains undergo substantial degradation. Within the temperature range of 430 to 600 °C, the final mass loss of the polymer reaches 87.13%. A small fraction of organic compounds continues to decompose, leaving behind mostly inorganic residues. Notably, at 240 °C, HDZ−A retains a residual mass of 91.74% without showing apparent signs of thermal decomposition. This indicates that HDZ−A exhibits excellent thermal stability and temperature resistance. The TG curve provides insights into the thermal decomposition behavior of the polymer at different temperatures. By analyzing the TG curve, the thermal stability and temperature resistance of HDZ−A can be evaluated. The results demonstrate that HDZ−A maintains relative stability even at elevated temperatures, which is of significant importance for its potential applications in high-temperature environments.

### 2.3. Optimization of Gel Plugging Agent Formulation

#### 2.3.1. Polymer Concentration Optimization

**Figure 3:**
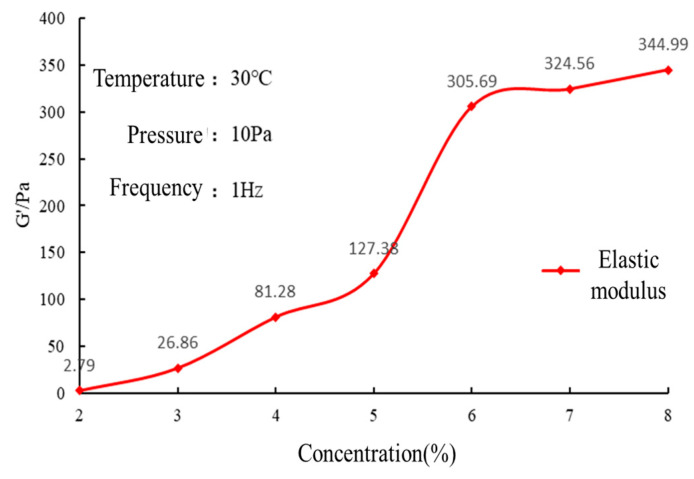
Experimental results of polymer concentration optimization.

The polymer concentration was designed to increase uniformly from 2 wt. % to 8 wt. %, while the crosslinking agent concentration was kept constant at 1.6 wt. %. The experimental results are shown in Figure 3.

With the increase in polymer concentration, the elastic modulus of the gel increased, but when the polymer concentration was 6 wt. %, the growth rate of the elastic modulus of the gel slowed down. The increase in polymer concentration leads to the increase in crosslinking sites between macromolecular chains and crosslinking agents, and the formation of three-dimensional network space decreases, resulting in the increase in gel strength. When the concentration increases to a certain extent, the crosslinking density between the polymer and the crosslinking agent decreases, and the excess macromolecular chain will exist in a free form. Although the strength increases, the amount of increase is small, and the initial viscosity will also increase significantly. Finally, the optimal polymer concentration is 6 wt. %.

The polymer HDZ−A is used as a thickener. The higher the concentration, the denser the crosslinking points between the macromolecular chain and the crosslinking agent, and the smaller the three-dimensional network space that is formed, meaning the gel strength will increase; when the concentration is too high, the crosslinking density between the polymer and the crosslinking agent will decrease, and the excess polymer molecular chain will exist in a free state. Although the strength still increases, the amount of increase is very small, and the initial viscosity will also increase significantly.

#### 2.3.2. Concentration Optimization of Crosslinking Agent

**Figure 4:**
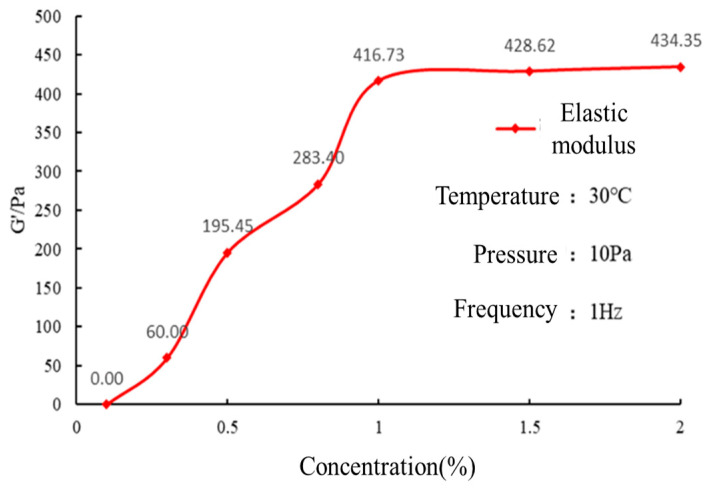
Experimental results of crosslinker concentration optimization with fixed 6% polymer concentration.

The polymer concentration was 6 wt. %, and the crosslinking agent concentration was designed to be 0.1 wt. %, 0.3 wt. %, 0.5 wt. %, 0.8 wt. %, 1 wt. %, 1.5 wt. %, 2 wt. %, a total of seven stages. The experimental results are presented in Figure 4.

At a crosslinking agent concentration of 0.1 wt. %, the polymer did not form a gel. As the concentration of the crosslinking agent increased, the elastic modulus gradually increased. However, once the concentration reached 1 wt. %, the gel strength no longer increased. Therefore, the optimal concentration of the crosslinking agent was determined to be 1 wt. %. The viscoelastic measurements were conducted to assess the effect of crosslinking agent concentration on the mechanical properties of the polymer. The results indicate a concentration-dependent increase in the elastic modulus until reaching a plateau at 1 wt. % crosslinking agent concentration. This concentration was determined to be the optimal choice for achieving the desired gel strength.

When the amount of crosslinking agent is low, the corresponding active monomer free radical concentration is low, the grafting rate between polymer molecular chains is limited, and a dense spatial crosslinking network chain structure cannot be formed, so the elastic modulus is small. With the increase in the amount of crosslinking agent, the grafting rate between the polymer molecular chains increases, and the crosslinking density of the polymer molecules increases, resulting in an increase in elastic modulus.

#### 2.3.3. Concentration Optimization of Retarder

**Figure 5:**
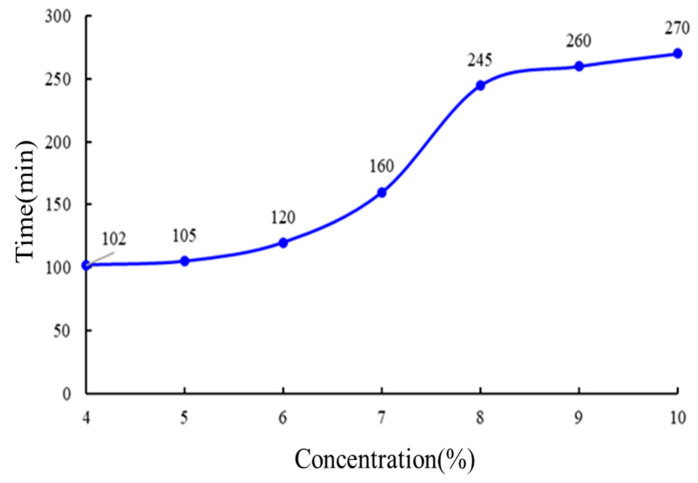
The experimental results of optimizing the concentration of the retarder by fixing the polymer concentration at 6% and the crosslinking agent concentration at 1%.

Gel solution preparation: 6 wt. % polymer HDZ−A + 1 wt. % crosslinking agent + retarder. The concentration of the retarder was designed to be 4 wt. %, 5 wt. %, 6 wt. %, 7 wt. %, 8 wt. %, 9 wt. % and 10 wt. %, respectively. The results are presented in Figure 5.

When the retarder concentration is low, the thickening time of the gel increases slowly, indicating that the low concentration of retarder has no significant retarding effect on the gel at high temperatures. When the concentration reaches 6 wt. %, the retarding time increases rapidly until the concentration is 8 wt. %, and the trend gradually slows down. The concentration of the retarder is 8 wt. %, which not only saves reagents, but also leads to a long thickening time. The retarder can consume part of the free radicals in the system, causing them to extend. This suggests that the gelation process can be controlled within a desired time range (1.5 to 4.5 h) by adjusting the concentration of the gelling agent at a temperature of 150 °C.

A preferred high-temperature-resistant and delayed polymer gel plugging system has been developed. The composition of the gel plugging system consists of 6 wt. % polymer, 1 wt. % crosslinking agent, and 8 wt. % gelling agent. The gel solution formulated with this recipe exhibits low-viscosity characteristics at room temperature. Furthermore, the rheological properties of the gel solution are minimally affected by temperature variations and shear rates. The delayed crosslinking time can reach up to 4.5 h.

### 2.4. Comprehensive Performance

#### 2.4.1. Structural Characteristics

**Figure 6:**
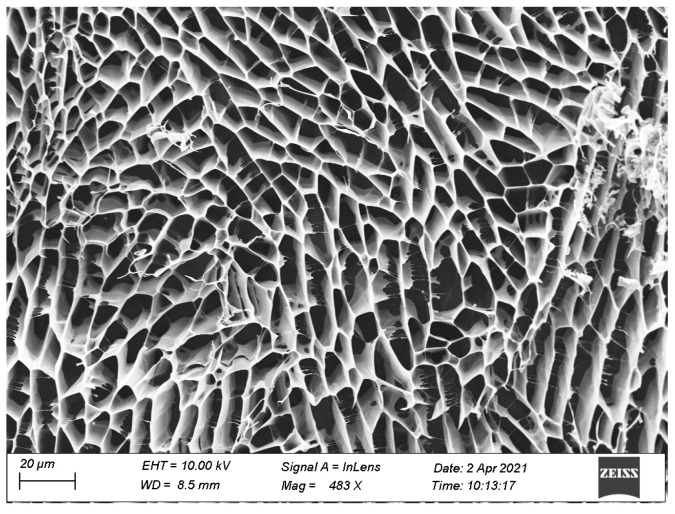
SEM image.

The gel plugging agent, after undergoing high-temperature crosslinking, was subjected to a drying process at 60 °C. The dried gel was then cut into well-defined shapes for scanning electron microscopy (SEM) testing. The experimental results are depicted in Figure 6.

From Figure 6, it is evident that the main backbone structure and branch structure of the gel plugging agent are clearly discernible. They support each other, forming an intertwined mesh structure. The gaps within each unit mesh are small, dense, and exhibit a regular distribution. Besides this, Figure 6 also reveals that the molecular chain structure of the gel plugging agent forms an intertwined multilayer structure resembling linear threads. Each layer constitutes a tree-like mesh structure, and the layers are interconnected through filamentous mesh structures. The gel plugging agent is composed of a three-dimensional network framework interwoven by multiple layers of tree-like structures, enhancing the structural strength of the gel plugging agent. The SEM analysis provides valuable insights into the microstructure of the gel plugging agent after high-temperature crosslinking and subsequent drying. The observed interlocking mesh structures, both at the macroscopic and molecular levels, contribute to the overall strength and stability of the gel plugging agent.

#### 2.4.2. Rheological Properties

**Figure 7:**
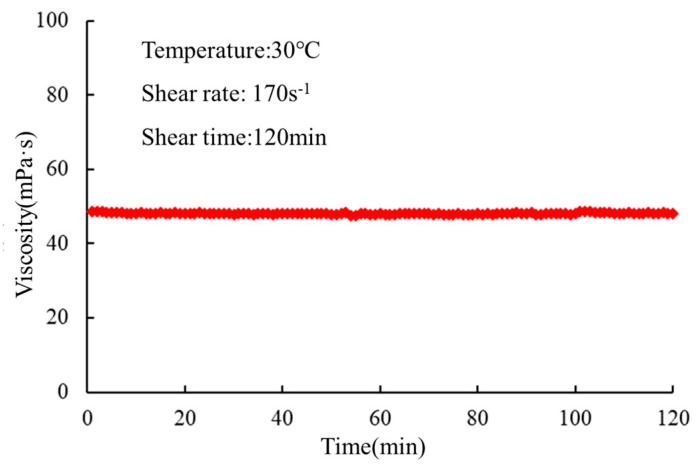
Rheological test at room temperature.

**Figure 8:**
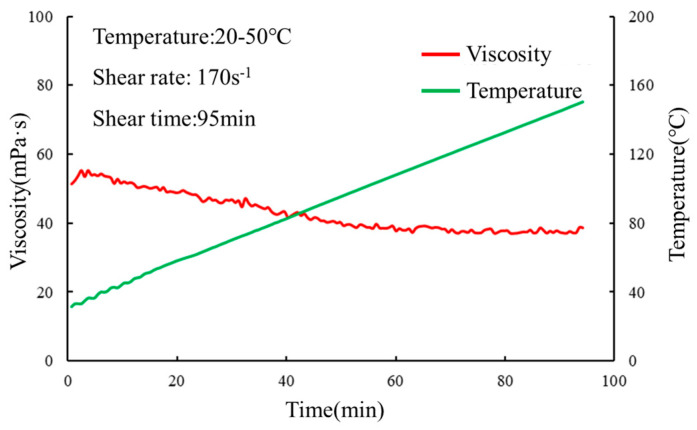
Rheological test at different temperatures.

The gel solution was subjected to rheological testing at both room temperature (30 °C) and variable temperatures (20 to 150 °C), and the results are presented in Figure 7 and Figure 8.

It can be observed that at both room temperature and variable temperatures, the gel solution exhibits low viscosity. Consequently, it requires low pumping pressure for transportation. The low viscosity of the gel solution reduces the likelihood of blockage within the wellbore, allowing it to penetrate deeply into the formation and enhance the plugging efficiency of deep fractures. The rheological properties of the gel solution were evaluated to assess its suitability for practical applications. The low viscosity of the gel solution enables easy handling and pumping, minimizing the risk of clogging during injection. This characteristic is particularly advantageous for reaching and effectively sealing deep fractures in the reservoir. Additionally, the gel solution’s low viscosity reduces the pumping pressure requirements, contributing to energy efficiency and cost-effectiveness during the deployment process.

#### 2.4.3. Temperature Resistance

**Figure 9:**
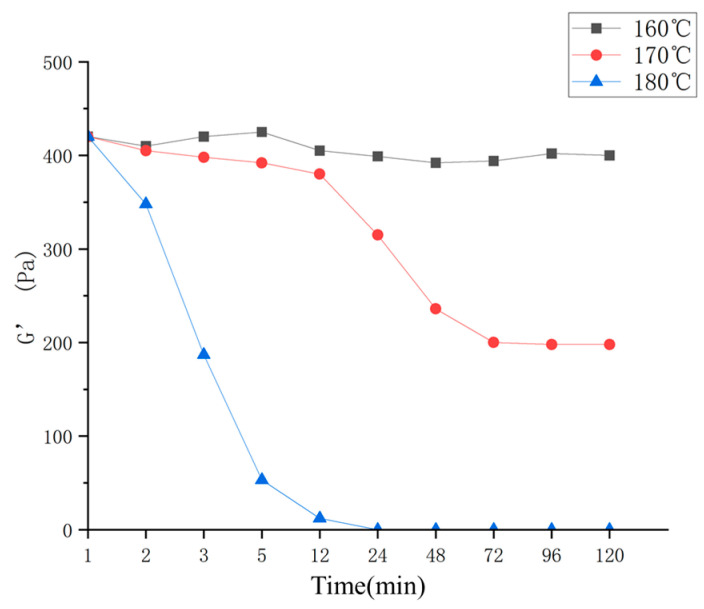
Test of temperature resistance.

High-temperature aging tests were conducted at temperatures of 160 °C, 170 °C, and 180 °C. The gel samples were subjected to rheological measurements at various time intervals, namely, 1 h, 2 h, 3 h 5 h, 12 h, 24 h, 48 h, 72 h, 96 h, 120 h, 144 h, and 168 h. The experimental results are depicted in Figure 9.

Based on Figure 9, it can be observed that at a temperature of 160 °C, the elastic modulus of the gel remains relatively constant. This indicates that the gel plugging agent is capable of effectively sealing fractures under the conditions of 160 °C. At a temperature of 170 °C, the elastic modulus exhibits a slight decrease during the initial 12 h, followed by a rapid decline. Eventually, the elastic modulus stabilizes at approximately half of its initial value, suggesting that the gel plugging material has limited temperature resistance at 170 °C and cannot provide long-term plugging of the reservoir. At a temperature of 180 °C, the elastic modulus experiences a significant decrease, and after 12 h, the gel loses its strength, rendering it unable to achieve effective plugging of the formation. At a temperature of 160 °C, the three-dimensional network structure of the gel plugging agent remains largely unaffected. At 170 °C, the molecular chains of the gel exhibit curling at their ends, leading to the disruption of some incompletely crosslinked, relatively unstable, and shorter polymer molecules. Despite the complete destruction of these molecules, the gel-plugging agent still retains a certain degree of strength. At 180 °C, the three-dimensional network structure of the gel plugging agent is completely destroyed, and the large molecular chains experience fractures, resulting in a rapid decline in strength until it eventually disappears.

#### 2.4.4. Delayed Crosslinking Property

**Figure 10:**
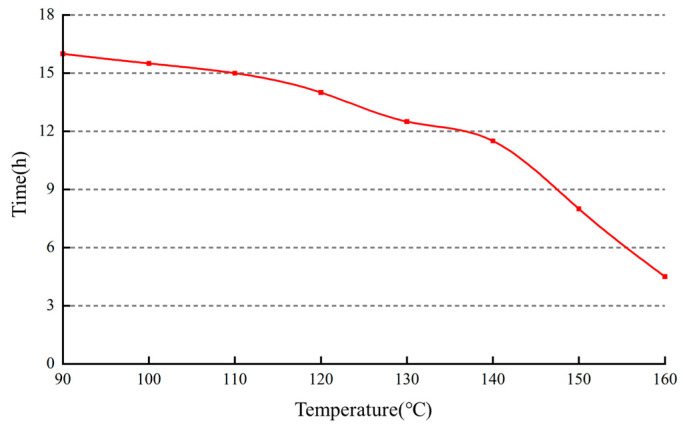
Effect of temperature on crosslinking time.

A high-temperature aging experiment was conducted by setting variable temperatures at 90 °C, 100 °C, 110 °C, 120 °C, 130 °C, 140 °C, 150 °C, and 160 °C. The aim was to observe the gelation time of the gel plugging agent at different temperatures, and the results are presented in Figure 10.

As the temperature increases, the gelation time progressively decreases. Beyond 140 °C, the reduction in gelation time becomes more pronounced. At a temperature of 160 °C, the gelation time reaches 4 h. Following this trend, it is projected that at 170 °C, the gelation time will decrease to approximately 1 h, indicating a minimal delay in gelation. The delayed crosslinking is achieved through the combined action of a crosslinking agent and a retarding agent. As the temperature rises to the critical temperature of the retarding agent, it begins to decompose, releasing the crosslinking agent. The crosslinking agent then interacts with the polymer to form the gel plugging agent. As the temperature increases, the decomposition rate accelerates. Beyond 140 °C, the retarding agent decomposes rapidly, leading to a significant reduction in gelation time. At temperatures above 160 °C, the crosslinking reaction occurs too quickly, resulting in a rapid decrease in gelation time. Consequently, it becomes unsuitable for the application of delayed crosslinking gel plugging agents.

### 2.5. Sealing Characteristics

#### 2.5.1. Salt Resistance

The gels were prepared using formation water with mineralization levels of 0 mg/L, 1000 mg/L, 5000 mg/L, 10,000 mg/L, 15,000 mg/L, and 20,000 mg/L (consisting of 25% KCl, 60% NaCl, 6% MgCl_2_, and 9% CaCl_2_).

**Figure 11:**
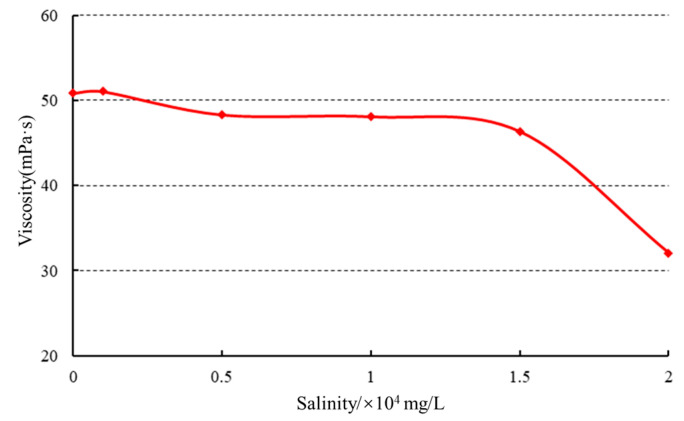
Effect of mineralization on initial viscosity.

(1) Influence of mineralization on initial viscosity. Rheological testing was performed on the gel solutions to examine the effect of mineralization on viscosity, as illustrated in Figure 11.

As the mineralization level increased, a slight decrease in viscosity was observed. However, once the mineralization reached 15,000 mg/L, the viscosity decreased rapidly. At lower mineralization levels, the polymer containing a substantial amount of negatively charged groups exhibited an extended conformation of molecular chains due to the electrostatic repulsion between polymer molecules. As the mineralization level increased, the positively charged metal ions neutralized the negative charges on the polymer chains, resulting in a reduction in or even absence of repulsive forces between the molecules. Consequently, the polymer chains underwent coiling and contraction, leading to a decrease in viscosity.

**Figure 12:**
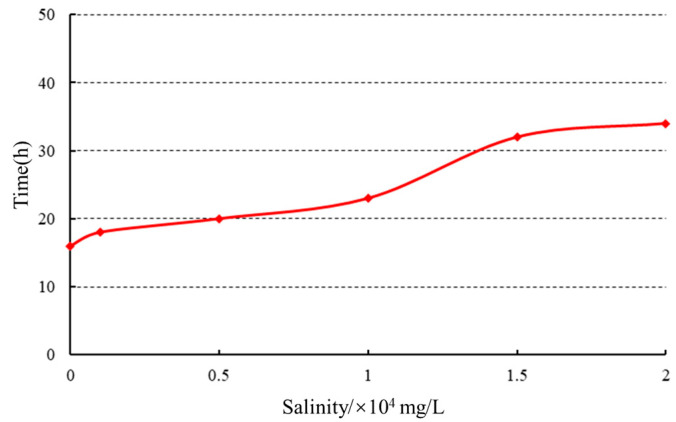
Effect of mineralization on gelation time.

(2) Influence of mineralization on gelation time. High-temperature aging experiments were conducted at 80 ℃ to investigate the effect of mineralization on gelation time, as depicted in Figure 12.

With increasing mineralization, the gelation time initially increased and then reached a plateau. The elevation of salt concentration intensified the salt-sensitive effect, resulting in the compression of the double electric layers around polymer chains. This led to the severe contraction and coiling of the polymer chain ends, a reduction in crosslinking sites, and a slower formation of the network structure, thus prolonging the gelation time. Once the mineralization level reached a critical value, the crosslinking sites reached their maximum limit, ultimately reaching the upper limit of gelation time.

**Figure 13:**
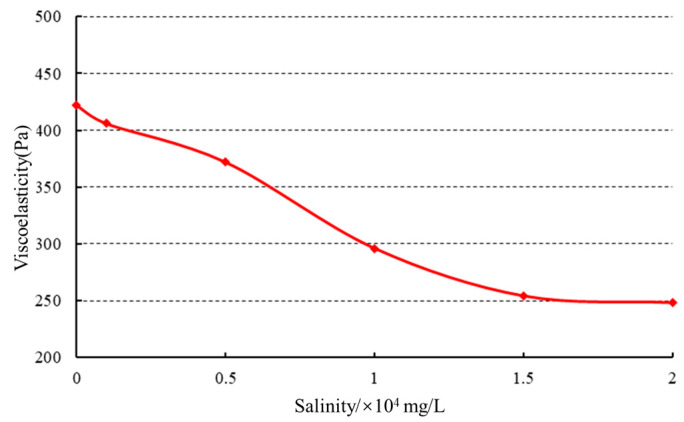
Effect of salinity on gel strength.

(3) Influence of mineralization on gel strength. Viscoelastic tests were performed, and the results are presented in Figure 13.

With increasing mineralization, the gel strength initially decreased and then remained constant. The increase in mineralization led to the coiling of polymer chains caused by salt sensitivity, resulting in a decrease in the density of crosslinking sites. This led to an enlargement of the spatial structure of the gel network and ultimately a reduction in gel strength. Once the mineralization level reached a critical value and continued to increase, the network structure became fixed, and the gel strength no longer changed.

#### 2.5.2. Sealing Characteristics

To evaluate the sealing performance of the product, quartz sand with different mesh sizes was selected and packed into sand-packed tubes, compacted appropriately. Simulated formation water (formulated as 2.0% KCl, 5.5% NaCl, 0.45% MgCl_2_, and 0.55% CaCl_2_) was used to test the permeability of the sand-packed tubes before and after crosslinking, and the plugging efficiency was calculated. The results are presented in Table 3. The gel plugging agent exhibited sealing efficiencies of 97.35% and 97.5% for quartz sands with mesh sizes of 40–60 and 20–40, respectively. The post-plugging permeabilities were measured as 0.241 mD and 0.512 mD, respectively.

#### 2.5.3. Pressure Bearing Capacity

In order to test the pressure bearing capacity of the plugging agent, rock samples with different fracture widths were prepared. The rock samples were put into the core plugging evaluator, and the pressure was increased from 0.5 MPa to 10 MPa. The cumulative filtration loss was calculated to determine the pressure bearing capacity of the gel plugging agent under different fracture widths. The experimental data are shown in Table 4.

According to Table 4, the rock samples with a crack width of 0.05 mm remained leak-free throughout the entire test duration as the pressure increased. The rock samples with a crack width of 0.1 mm started to experience filtration loss when the pressure reached 10 MPa, with a total cumulative loss of 0.13 mL. The rock samples with a crack width of 0.2 mm exhibited relatively stable filtration loss within the range of 7 MPa to 10 MPa. For the rock samples with a crack width of 0.5 mm, leakage began at 7 MPa, and as the pressure increased to 10 MPa, the cumulative loss increased from 0.108 mL to 2.428 mL. The rock samples with crack widths of 1 mm and 2 mm started to leak at a pressure of 6 MPa, with cumulative losses reaching 9.655 mL and 8.766 mL, respectively. The rock samples with a crack width of 5 mm began to leak at 5 MPa, and the cumulative loss reached 11 mL by 9 MPa. The pressure-bearing capacity reached above 9 MPa when the crack width was less than 0.1 mm, between 6 and 7 MPa when the crack width was less than 2 mm, and 4 MPa for a crack width of 5 mm.

### 2.6. Mechanism of Action

(1) Thermal Resistance Mechanism

With increasing temperature, the properties of functional groups on the gel polymer chains undergo changes, and the molecular chains contract and curl at the ends. Additionally, the connection between the crosslinking agent and the polymer may break, resulting in a reduction in the crosslinking density of the gel plugging agent. This leads to an increase in the spatial size of the three-dimensional network structure and a weakening of its spatial integrity. If all crosslinking bonds are broken, the gel plugging agent will decompose into individual polymers, rendering the plugging agent ineffective.

(2) Delayed Crosslinking Mechanism

The delayed crosslinking effect is primarily achieved through the chelation of the retarder with the crosslinking agent, and the retarder is mainly influenced by temperature. When the temperature reaches the critical point of the retarder, it gradually decomposes, releasing the crosslinking agent, which interacts with the polymer to form a plugging gel. Higher temperatures result in faster decomposition rates of the retarder, leading to poorer delayed crosslinking effects. In this study, the retarder used in the gel plugging agent exhibited a significant increase in decomposition rate when it reached 140 °C, reducing the delayed crosslinking performance. Beyond 160 °C, the retarder decomposes rapidly, making it unable to achieve the desired delayed crosslinking effect.

(3) Salt Resistance Mechanism

Since the polymer is primarily composed of acrylamide, the resulting product contains a large number of negatively charged groups. When the salinity is low, the electrostatic repulsion between the polymer chains keeps them in an extended state. However, when the salinity reaches 15,000 mg/L, the metal cations neutralize the negative ions carried by the polymer, weakening or even eliminating the repulsive force between the molecular chains. As a result, the polymer chains contract and curl, leading to a decrease in initial viscosity.

As the salt concentration increases, the salt-induced effect strengthens, compressing the double electric layers around the polymer chains. This causes the severe contraction and curling of the polymer chains at the ends, resulting in a reduction in the number of crosslinking sites between the polymer chains and the crosslinking agent. Consequently, the formation of the network structure slows down, and the gelation time increases. Once the salt concentration reaches a certain threshold, the connection sites between the polymer chains and the crosslinking agent reach their maximum, causing the rate of gelation time increase to slow down. With further increases in salinity, the ultimate gelation time reaches a threshold value.

### 2.7. Field Application

In order to test the actual effectiveness of the gel plugging agent, a field experiment was conducted in well KT−14X in the Tianhuan Depression of the Ordos Basin. Prior to the field experiment, well KT−14X experienced multiple instances of leakage, with a total loss of drilling fluid exceeding 2000 m^3^.

(1) Plugging Operation Process

A wellbore leak occurred at a depth of 3400 m in well KT−14X. Various methods such as drilling fluid loss prevention while drilling, fiber cement slurry plugging, high-loss water-based mud plugging, and bridging slurry + cement and bridging plugging were used multiple times, but the results were not satisfactory as the leaks reoccurred after a certain period of drilling. Subsequently, an operation using a high-temperature-resistant delayed crosslinking polymer gel plugging system was employed for plugging. During the operation, various materials were added according to the formulation. HDZ−A was slowly and uniformly added and thoroughly stirred. A bridging slurry of 20–30 m^3^ was prepared, with the specific dosage determined based on the leak rate. If the leak rate was >10 m^3^/h, 10–15 m^3^ of bridging slurry was pumped in before injecting HDZ−A gel. If the leak rate was ≤10 m^3^/h, HDZ−A gel was directly injected. A cementing pump with a displacement of 11 L/s was used to pump in 10 m^3^ of HDZ−A gel, followed by the injection of 10–15 m^3^ of bridging slurry after the gel plugging agent was injected. Finally, the gel plugging agent and bridging slurry were replaced with drilling fluid, and based on the intake of HDZ gel and the squeeze pressure, efforts were made to inject HDZ−A gel into the leak zone. After the completion of the squeeze, drilling was resumed to a safe interval or within the casing, with the first few stands drilled slowly to prevent backflow of the plugging slurry during circulation, followed by an 8 h waiting period.

(2) Evaluation of Plugging Effectiveness

After the waiting period, drilling resumed with a PDC drill bit, and circulation was conducted in sections. The following day, drilling continued to a depth of 3400 m with normal circulation. The density was increased to 1.14 g/cm^3^, and normal returns were observed at normal flow rates without any loss. Drilling continued to 3733 m, with normal formation cuttings and mud consumption, and a density of 1.14 g/cm^3^. Drilling proceeded to a depth of 3795 m, and mud consumption remained normal during drilling. This indicates that the HDZ−A gel system had a good plugging effect, and no reoccurrence of complex downhole conditions was observed, thus achieving the objective of the plugging operation.

## 3. Conclusions

(1) A high-temperature- and salt-resistant polymer, HDZ−A, was developed, and the optimal synthesis conditions were determined. The optimal conditions were as follows: monomer concentration of 8 wt. %, initiator concentration of 0.3 wt. %, synthesis temperature of 70 °C, and monomer molar ratio of AM:AMPS:ACMO = 7:1.75:1.25.
(2) Using the newly developed high-temperature- and salt-resistant polymer, HDZ−A, as a key treatment agent, a formula for an anti-high-temperature delayed crosslinking polymer gel plugging system was optimized. The specific formulation consists of 6 wt. % polymer HDZ−A, 1 wt. % crosslinking agent, and 8 wt. % retarder. This gel plugging system can withstand high-temperature reservoirs up to 160 °C, and the gelation time at 160 °C can reach a maximum of 4.5 h.
(3) Field applications of the newly developed anti-high-temperature delayed crosslinking polymer gel plugging system were assessed. The results of the field experiments show that the construction process of the new gel plugging system was simple. After using this gel plugging system for plugging operations, no reoccurrence of complex downhole conditions was observed, and the consumption of drilling fluid during the drilling process after plugging was normal, indicating no further loss. The plugging effect was significant.

## 4. Materials and Methods

### 4.1. Materials

Acrylamide (AM) of chemically pure grade was obtained from the China National Pharmaceutical Group Chemical Reagent Co., Ltd in Shanghai, China. 2-Acrylamido-2-methylpropane sulfonic acid (AMPS) of chemically pure grade was recrystallized with acetic acid and obtained from Aladdin Reagent in Shanghai, China. N-Acryloylmorpholine (ACMO) of chemically pure grade was obtained from Aladdin Reagent in Shanghai, China. Thirty percent hydrogen peroxide of chemical pure grade was obtained from Millipore Reagent in Shanghai, China. Alkaline chromium acetate of chemically pure grade was obtained from Aladdin Reagent in Shanghai, China. Sodium lactate of chemically pure grade was obtained from Aladdin Reagent in Shanghai, China.

### 4.2. Synthesis Principle of HDZ−A

A high-temperature-resistant delayed crosslinking gel plugging agent is mainly composed of three parts: polymer, crosslinking agent, and retarder. As the main body of a gel plugging agent, the performance of the polymer determines the performance of the gel plugging agent.

AM has good water solubility and high chemical activity, and it is easy to obtain a variety of modifiers with branched or network structures by grafting or crosslinking. Therefore, an acrylamide monomer is selected as the main chain in this paper. Because the monomer AMPS contains a sulfonic acid group, a polymerizable vinyl group, and a ahydrophilic sulfonic acid group, it can be copolymerized with a variety of water-soluble and non-water-soluble monomers. At the same time, the sulfonic acid group has good temperature resistance and salt resistance. The morpholine group of ACMO has the characteristics of fast curing, low viscosity, and strong dilution ability. It is introduced into the polymer to enhance the gel properties of the polymer gel plugging system. Therefore, in this paper, a new polymer HDZ−A was synthesized with free radical aqueous solution polymerization using AM, AMPS, and ACMO as the main raw materials.

**Figure 14:**
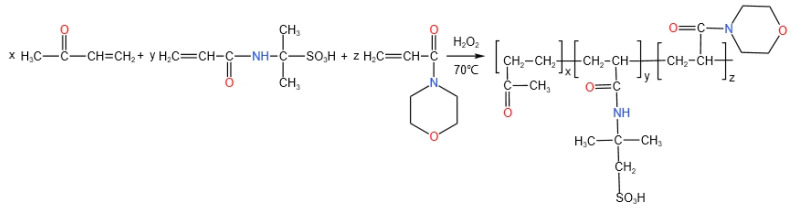
Synthesis principle of HDZ−A.

AM, AMPS, and ACMO form a new type of polymer HDZ−A by free radical aqueous solution polymerization. Firstly, in the presence of an initiator, the chain initiation reaction is carried out to form a free radical active center, and the initiator decomposes to generate a primary free radical. After the formation of monomer free radicals, the copolymerization continues with other monomers, and the reaction enters the chain growth stage. The monomer free radicals produced in the chain initiation stage have high reactivity, opening the π bond of the second hydrocarbon monomer, rehybridizing, and combining to form a new free radical. The activity of the new free radical does not decay, and it continues to bind to other monomer molecules to form more chain free radicals containing monomer units, thereby completing the chain growth reaction of chain free radicals. A prominent feature of AM polymerization is that the growing chain is highly hydrated in an aqueous solution. This hydration layer significantly shields the double-base termination probability of the growing radical, which greatly prolongs the life of the chain radical, so that a product with a high degree of polymerization can be obtained. The synthesis principle of HDZ−A is illustrated in Figure 14.

### 4.3. Synthesis of HDZ−A

In total, 14 g of acrylamide monomer was accurately weighed and dissolved in 50 mL of distilled water; 3 g of 2-acrylamido-2-methylpropane sulfonic acid (AMPS) was weighed and fully dissolved in 50 mL of distilled water. Additionally, 3 g of N-acryloylmorpholine (ACMO) was weighed and dissolved in 50 mL of distilled water. The three solutions were then poured into a three-neck flask, and 0.6 mL of hydrogen peroxide was accurately measured and dissolved in 50 mL of water. The hydrogen peroxide solution was then transferred into a constant-pressure dropping funnel. The flasks containing the three solutions and the dropping funnel were assembled. The mixture was stirred and heated to 35 °C while purging with nitrogen gas for 30 min to remove oxygen. The temperature was then raised to 95 °C. The stopcock of the dropping funnel was opened, and the solution was added dropwise at a controlled rate of 10–15 min. The reaction proceeded for 3 h. After the reaction, the polymer HDZ−A was obtained and transferred to a container for further use.

### 4.4. Evaluation of HDZ−A

The gel formulation was evaluated as follows: 6.4 g HDZ−A + 1.6 g crosslinking agent + 100 g water (6.4 wt. % HDZ−A + 1.6 wt. % crosslinking agent). The crosslinking agent was alkaline chromium acetate, and the retarder was sodium lactate.

The HAKKE MARS III rheometer was used to determine the viscosity of the uncrosslinked gel solution. The experimental conditions were set as 170 s^−1^, with a temperature of 30 °C, and a test time of 20 min.

The gel solution was prepared at room temperature. After testing its viscosity, the gel solution was placed in a high-temperature reactor, heated to 150 °C, crosslinked at high temperature for 1 h, and cooled after gelation. After the gel was cooled to room temperature, 5 mL was taken out, and the viscoelasticity test was carried out using a HAKKE MARS III rheometer. The experimental conditions were set as follows: 30 °C, fixed scanning frequency 1 Hz, and stress 10 Pa. The viscoelasticity of the gel can be used to characterize the dynamic modulus of the gel, including the G’ elastic modulus and the G’’ viscous modulus.

### 4.5. Characterization of HDZ−A

The molecular structure of HDZ−A was characterized by the KBr tablet pressing method using a Nexus Fourier transform infrared spectrometer. The ZNN−D6 six-speed rotary viscometer was used to measure the apparent viscosity of the HDZ−A solution. The microstructure of HDZ−A in aqueous solution was observed with a Quanta 450 environmental electron scanning microscope by the freeze sublimation method. The thermal stability of HDZ−A was investigated by TG analysis. The experimental temperature range was 0~600 °C, and the heating rate was 5 °C/min.

### 4.6. Performance Evaluation of Polymer

(1) Preparation of gel solution: The gel formulation consisted of 6.4 wt. % polymer, 1.6 wt. % crosslinking agent, and 5 wt. % retarder.
(2) Rheological testing: The viscosity of the gel solution before crosslinking was measured using a HAKKE MARS III rheometer at a temperature of 30 °C, shear rate of 170 s^−1^, and testing time of 20 min. The viscosity of the gel solution before crosslinking reflects its rheological properties during the pumping process into the formation. A lower viscosity indicates lower pumping pressure and a lower probability of retention in the wellbore.
(3) High-temperature crosslinking: The gel solution was prepared at room temperature, and its viscosity was measured. The gel solution was then placed in a high-temperature reaction vessel and heated to 150 °C for 1 h for high-temperature crosslinking. After gelation, it was removed and cooled. The crosslinking status of the gel was visually observed during this process to evaluate its crosslinking performance.
(4) Viscoelastic testing: The prepared gel solution was subjected to high-temperature crosslinking in the reaction vessel. After a certain temperature and time, the elastic modulus (G’) of the gel was measured using a rheometer at a temperature of 30 °C, shear stress of 10 Pa, and frequency of 1 Hz. The average elastic modulus was calculated. For polymer gels, a higher elastic modulus indicates higher strength and better crack sealing performance.

## Figures and Tables

**Table 1:** Factor level table of the orthogonal test.

Levels	Factor			
	A	B	C	D
	Concentration/wt. %	Mass Fraction of Initiator/wt. %	Temperature/°C	Monomer Molar Ratio AM:AMPS:ACMO
1	8	0.3	60	7:1.25:1.75
2	9	0.35	65	7:1.5:1.5
3	10	0.4	70	7:1.75:1.25

**Table 2:** Orthogonal test table of HDZ-A.

	A	B	C	D	µ/mPa·s	G’/Pa	X	Y	H
1	A1	B1	C1	D1	50.87	282.29	1.000	0.999	−0.001
2	A1	B2	C2	D2	25.21	263.68	0.090	0.899	0.809
3	A1	B3	C3	D3	25.90	269.50	0.115	0.931	0.816
4	A2	B1	C3	D2	33.41	282.44	0.381	1.000	0.619
5	A2	B2	C1	D3	22.67	188.03	0	0.494	0.494
6	A2	B3	C2	D1	35.24	130.99	0.446	0.188	−0.258
7	A3	B1	C2	D3	23.93	158.95	0.045	0.338	0.293
8	A3	B2	C3	D1	37.08	111.55	0.511	0.084	−0.427
9	A3	B3	C1	D2	31.87	95.95	0.326	0	−0.326
K_1_	0.902	0.749	0.235	−0.229					
K_2_	0.593	0.546	0.395	0.367					
K_3_	0.229	0.428	0.427	0.534					
R	0.673	−0.32	0.193	0.763					
optimal condition	A1	B1	C3	D3					

**Table 3:** Sealing performance test results.

Mesh	Permeability before Sealing K_1_/mD	Permeability after Sealing K_2_/mD	η/%
40~60	9.084	0.241	97.35
20~40	17.1	0.512	97.005

**Table 4:** Pressure bearing capacity test results.

Pressure/MPa	Cumulative Leakage/mL						
	0.05 mm	0.1 mm	0.2 mm	0.5 mm	1 mm	2 mm	5 mm
4	0	0	0	0	0	0	0
5	0	0	0	0	0	0	0.792
6	0	0	0	0	0.255	0.95	1.269
7	0	0	0.041	0.108	0.587	2.822	1.448
8	0	0	0.043	0.653	3.804	6.05	8.021
9	0	0	0.046	1.305	9.655	8.766	11.036
10	0	0.13	0.512	2.428			

## Data Availability

The raw/processed data required to reproduce these findings cannot be shared at this time as the data also form part of an ongoing study.
